# Dose–response of inactivated yeast in diets of late gestating and lactating gilts on immunoglobulin transfer and offspring preweaning growth performance

**DOI:** 10.1093/jas/skae177

**Published:** 2024-07-05

**Authors:** Brenda Christensen, Hagen Schulze, Elijah G Kiarie, Lee-Anne Huber

**Affiliations:** Department of Animal Biosciences, University of Guelph, Guelph, Ontario, CanadaN1G 2W1; Livalta, AB Agri Ltd., Peterborough, Cambridgeshire PE2 6FL, UK; Department of Animal Biosciences, University of Guelph, Guelph, Ontario, CanadaN1G 2W1; Department of Animal Biosciences, University of Guelph, Guelph, Ontario, CanadaN1G 2W1

**Keywords:** immunoglobulin transfer, inactivated yeast, neonatal pig, reproductive gilt

## Abstract

Fifty gilts (initial body weight [BW] 190.7 ± 4.2 kg) were recruited on day 85 of gestation and were used until day 19 of lactation to assess the dose–response of inactivated yeast via hydrolyzation (**HY**) inclusion on offspring growth and immunoglobulin (**Ig**) transfer prior to weaning. Gilts were assigned to one of the 5 experimental diets: a control with no HY (**HY0**) or inclusion of 0.25% (**HY0.25**), 0.5% (**HY0.5**), 1.0% (**HY1.0**), or 1.2% (**HY1.2**) HY. Gilts were weighed on days 85 and 110 of gestation and days 1 and 19 (weaning) after farrowing. Offspring were weighed on days 1 and 19 of age. On lactation day 1 (approximately 24 h after farrowing), colostrum, gilt plasma, and plasma from 2 median BW piglets were collected and on day 19, plasma from each gilt and 2 median BW piglets per litter were collected for determination of Ig concentrations. Contrast statements were used to assess the linear, quadratic, cubic, and quartic effects of HY inclusion. The inclusion of HY had minimal effects on gilt BW or litter characteristics at birth (total number born and born alive, piglet birth weight). Lactation average daily feed intake of the gilts tended to increase then decrease with increasing HY inclusion (quadratic; *P *= 0.085). Piglet preweaning average daily gain (linear, quadratic, and quartic; *P *< 0.05) and BW at weaning (quadratic and quartic; *P *< 0.05) increased then decreased with increasing HY inclusion. On lactation day 1, colostrum and gilt plasma Ig concentrations were not affected by dietary treatment (*P *> 0.10) but piglet IgA and IgM decreased then increased with HY inclusion level (cubic; *P *< 0.05). On lactation day 19, piglet plasma IgG tended to increase with HY inclusion (linear; *P *= 0.099). In summary, increasing HY inclusion in late gestating and lactating gilt diets improved immune transfer in the first 24 h after birth and piglet preweaning growth rates and BW at weaning. Therefore, maternal feeding of HY could be used as a strategy to improve offspring immunocompetence and BW at weaning, with possible carryover benefits for the postweaning phase.

## Teaser

Piglet body weight at weaning and immunoglobulin A and M concentrations after birth were positively related to inactivated yeast inclusion in the diets of gestating and lactating gilts, which is expected to improve piglet growth performance directly following weaning, without the use of in-feed growth promoters.

## Introduction

Weaning exposes piglets to various stressors that result in a period after weaning with minimal body weight (BW) gain or feed intake and increased incidence of morbidity and mortality. Low and variable feed intakes immediately after weaning contribute to the inconsistent beneficial effects of growth-promoting feed additives beyond antibiotics and pharmacological inclusion (>150 ppm) of zinc oxide (Wensley et al., 2021). In addition, concerns regarding the development of antibiotic-resistant bacteria and environmental sustainability have led to implementation of legislation restricting or banning the use of in-feed antibiotics and pharmacological inclusion of zinc oxide (EMA, 2017). Therefore, novel approaches to expedite the immunological transition of piglets at weaning are required for pig production.

The immunoglobulins (**Ig**) present in colostrum are essential for piglets to achieve passive immunity from the sow since piglets are born agammaglobulinemic (Salmon et al., 2009). Colostrum contains very high concentrations of IgG, but IgA becomes the main Ig in milk (i.e., mammary secretions occurring beyond 4 d after parturition; Hurley and Theil, 2011; Loisel et al., 2013). Reduced IgA concentrations in mammary sections of sows have been linked to higher incidence of preweaning piglet diarrhea and mortality (Amatucci et al., 2022), while gilts have lower Ig concentrations than sows, which reduces immunocompetence of the offspring and increases vulnerability toward subsequent immune challenge (Forner et al., 2021). Therefore, improving passive immunity, especially for the offspring of gilts, could be a strategy to help mitigate the postweaning growth lag and associated morbidity.

Yeast is a feed additive that provides both nutrients and bioactive compounds (i.e., β-glucans and mannan oligosaccharides; Anwar et al., 2017) and can be provided in various forms (i.e., probiotic, prebiotic, postbiotic, or combinations thereof) depending on processing methods (as reviewed by Patterson et al., 2023). Since yeast is an immune-modulating additive, it has been shown to reduce growth in nursery pigs when included in high quantities, likely due to over-stimulation of the immune system (Li et al., 2006; Anwar et al., 2017). Yeast-dependent stimulation of the immune system could, however, promote increased antibody production in reproductive animals and subsequent passive immune transfer to the offspring via colostrum (Hasan et al., 2018). Therefore, it was hypothesized that providing inactivated yeast (**HY**; enzymatically hydrolyzed *Saccharomyces cerevisiae*) to late gestating and lactating gilts would improve passive immune transfer to offspring prior to weaning. The objective of this study was to assess the dose–response of HY inclusion in the maternal diet on offspring growth performance and immunoglobulin (**Ig**) transfer prior to weaning.

## Materials and Methods

The experimental protocol was approved by the University of Guelph Animal Care Committee and followed Canadian Council on Animal Care guidelines (CCAC, 2009).

### Animals and diets

A total of 50 Yorkshire and Yorkshire × Landrace gilts (initial body weight [**BW**] 190.7 ± 4.2 kg) were recruited for the study on day 85 of gestation over 3 breeding batches and were fed experimental diets until weaning. Gilts were assigned to the respective dietary treatment based on day 85 BW, expected farrowing date, and breed (*n* = 1 to 2 Yorkshire and *n* = 8 to 9 Yorkshire × Landrace per dietary treatment) and received the same assigned HY inclusion level in both gestation and lactation. Dietary treatments for the gestating and lactating phases were: 0% (**HY0**), 0.25% (**HY0.25**), 0.5% (**HY0.5**), 1.0% (**HY1**), or 1.2% (**HY1.2**; *n* = 10; Table 1). The HY was enzymatically hydrolyzed *S. cerevisiae* which contained 32% crude protein and 40% β-1.3/1.6-glucans and mannooligosaccharides (Livalta TMCell HY40, AB Agri Ltd., Peterborough, UK). All diets were formulated to meet or exceed by NRC (2012) estimated nutrient requirements within phase (Table 1). Diet formulation considered the estimated energy, protein, and amino acids provided by HY. Between days 85 and 110 of gestation, gilts were given 2.6 kg of the assigned gestation diet per day. Between day 110 and farrowing, 2 kg of the assigned lactation diet was given per day, and after farrowing, a 3-d step-up program was used after which feed was provided ad libitum. Creep feed was not offered to the offspring and gilts were weaned on day 19.3 ± 0.2 of lactation, which constituted the end of the experiment.

**Table 1.** Ingredient composition and calculated and analyzed nutrient contents of gestation and lactation diets (as-fed basis)^1^

	Gestation					Lactation				
Item	HY0	HY0.25	HY0.5	HY1.0	HY1.2	HY0	HY0.25	HY0.5	HY1.0	HY1.2
*Ingredient, %*										
Corn	38.6	38.5	38.4	38.1	38.1	44.4	44.3	44.2	44.0	43.9
Soybean meal	8.8	8.7	8.6	8.4	8.3	25.6	25.5	25.4	25.1	25.0
Barley	42.0	42.0	42.0	42.0	42.0	10.0	10.0	10.0	10.0	10.0
Wheat	5.0	5.0	5.0	5.0	5.0	12.5	12.5	12.5	12.5	12.5
Fat, animal vegetable blend	1.0	1.0	1.0	1.0	1.0	3.0	3.0	3.0	3.0	3.0
Limestone	1.5	1.5	1.5	1.5	1.5	1.6	1.6	1.6	1.6	1.6
Monocalcium phosphate	1.4	1.4	1.4	1.4	1.4	1.6	1.6	1.6	1.6	1.6
Sodium chloride	0.5	0.5	0.5	0.5	0.5	0.5	0.5	0.5	0.5	0.5
Vitamin and trace minerals premix^2^	0.6	0.6	0.6	0.6	0.6	0.6	0.6	0.6	0.6	0.6
l-Lysine HCl	0.33	0.31	0.29	0.25	0.24	0.09	0.08	0.06	0.03	0.03
l-Threonine	0.19	0.18	0.17	0.15	0.15	—	—	—	—	—
dl-Methionine	0.05	0.06	0.06	0.06	0.06	0.01	0.01	0.01	—	—
l-Valine	0.02	0.02	0.02	—	—	0.04	0.03	0.02	—	—
l-Tryptophan	0.01	0.01	—	—	—	—	—	—	—	—
HY^3^	—	0.25	0.50	1.00	1.20	-	0.25	0.50	1.00	1.20
Total	100.0	100.0	100.0	100.0	100.0	100.0	100.0	100.0	100.0	100.0
*Calculated nutrient composition* ^4^										
Net energy, kcal/kg	2,415	2,414	2,413	2,412	2,411	2,500	2,499	2,499	2,497	2,497
Crude protein, %	13.20	13.20	13.20	13.20	13.20	18.51	18.51	18.51	18.51	18.51
SID Lys, %	0.80	0.80	0.80	0.80	0.80	1.03	1.03	1.03	1.03	1.04
SID Met + Cys, %	0.54	0.54	0.55	0.56	0.56	0.63	0.64	0.64	0.64	0.64
SID Thr, %	0.63	0.63	0.63	0.63	0.63	0.68	0.69	0.70	0.72	0.73
SID Trp, %	0.15	0.15	0.15	0.15	0.15	0.23	0.23	0.24	0.25	0.25
SID Ile, %	0.48	0.48	0.49	0.51	0.51	0.75	0.76	0.77	0.79	0.80
SID Val, %	0.61	0.62	0.63	0.63	0.63	0.89	0.89	0.89	0.89	0.90
Calcium, %	0.98	0.98	0.97	0.97	0.97	1.10	1.10	1.10	1.10	1.10
STTD phosphorus, %^5^	0.38	0.38	0.38	0.38	0.38	0.47	0.47	0.47	0.47	0.47
Total phosphorus, %	0.63	0.63	0.63	0.63	0.63	0.72	0.72	0.72	0.72	0.72
*Analyzed composition*										
Gross energy, kcal/kg	3,808	3,805	3,746	3,818	3,810	3,947	3,916	3,939	3,912	3,926
Crude protein, %	13.47	12.09	12.34	12.33	12.09	17.18	17.65	17.85	17.36	17.39
Calcium, %	0.90	0.86	0.81	0.79	0.79	0.97	0.98	0.94	1.05	0.96
Phosphorus, %	0.62	0.57	0.58	0.56	0.54	0.62	0.66	0.61	0.62	0.65

^1^Dietary treatments: HY0, control gestation and lactation diets; HY0.25, HY0.5, HY1.0, and HY1.2 included either 0.25%, 0.5%, 1.0% or 1.2%, respectively, of yeast inactivated via hydrolyzation. Diets were fed between day 85 of gestation and day 19 of lactation (weaning).

^2^Provided per kilogram of premix: vitamin A, 2,000,000 IU as retinyl acetate; vitamin D3, 200,000 IU as cholecalciferol; vitamin E, 8,000 IU as dl-α-tocopherol acetate; vitamin K, 500 mg as menadione; pantothenic acid, 3,000 mg; riboflavin, 1,000 mg; choline, 100,000 mg; folic acid, 400 mg; niacin, 5,000 mg; thiamin, 300 mg; pyridoxine, 300 mg; vitamin B12, 5,000 mcg; biotin, 40,000 mcg; Cu, 3,000 mg from CuSO_4_ × 5H_2_O; Fe, 20,000 mg from FeSO_4_; Mn, 4,000 mg from MnSO_4_; Zn, 21,000 mg from ZnO; Se, 60 mg from Na_2_SeO_3_; and I, 100 mg from KI (Grand Valley Fortifiers, Cambridge, ON, Canada).

^3^Enzymatically treated whole non-GMO *S. cerevisiae* strain assayed to contain 40% β-1.3/1.6 glucans and mannan oligosaccharides and 36% crude protein (Livalta TMCell HY40, AB AGRI, Peterborough, UK).

^4^Calculated based on the NRC (2012) ingredient values.

^5^Standardized total tract digestible phosphorus.

### Experimental procedures and samples collection

Individual gilt BW was recorded on days 85 and 110 of gestation, on day 1 after farrowing, and at weaning. Feed refusal was recorded daily in gestation and total feed intake was recorded during the lactation period. Within 24 h of parturition, piglets were weighed, and litters were standardized to 12 piglets. Approximately 24 h after the birth of the first piglet, colostrum was collected into a sterile 50-mL conical tube from all functional teats on the right side (approximately 6 mL per teat) and stored at −20 °C until further analysis. The precise interval between delivery of the first piglet and colostrum collection was determined through video recordings (Canon Vixia RF800; Canon Canada Inc., Brampton, ON, Canada).

Immediately following colostrum collection and at weaning, blood samples from the gilt (10 mL) and 2 median BW piglets (5 mL) were collected through the retro-orbital sinus (Dove and Alworth, 2015) using a Monoject Standard Hypodermic needle (16 G × 1.5ʹʹ and 18 G × 1.5ʹʹ, respectively; Covidien, Mansfield, MA) into vacutainer tubes coated with lithium heparin (Becton Dickinson & Co, Franklin Lakes, NJ). Once collected, samples were allowed to cool for 30 min before being centrifuged at 1,400 × *g* for 20 min at room temperature. After centrifugation, plasma was aliquoted into 1.5 mL microcentrifuge tubes and stored at −20 °C until further analysis. At weaning, piglets were weighed again, and fresh, sterile fecal samples were collected from each gilt, immediately placed in liquid nitrogen, and then stored at −80 °C until further analyses.

### Sample processing and laboratory analyses

Subsamples of each batch of gestation and lactation diet were collected and stored at 4 °C until analysis. Samples were finely ground (Custom Grind Coffee Grinder, Hamilton Beach Brands Canada Inc., Belleville, ON, Canada), then analyzed for dry matter (AOAC, 2005; method 930.15), crude protein (AOAC, 2005; method 968.06), calcium, phosphorus, potassium, and magnesium using inductively coupled plasma mass spectrophotometry (AOAC, 2005; method 985.01) and sodium using inductively coupled plasma-optical emission spectrometry (AOAC, 2005; method 2011.14; Agrifood Laboratories, Guelph, ON, Canada).

Plasma and colostrum samples were analyzed for IgA, IgG, and IgM using Bio-Rad antibodies (Bio-Rad Laboratories, Mississauga, ON, Canada) on a high-binding 384-well plate (Corning, Acton, MA). Prior to analysis, colostrum was thawed and delipidated according to Srijangwad et al. (2021). The protocol from Begley et al. (2009) was followed for IgG analysis, with adjustments made for IgA and IgM analysis. In brief, rabbit anti-pig IgG was used as a primary antibody (1:35,000), samples were diluted 1:500 with 0.05% phosphate-buffered saline with tween-20 (**PBST**) buffer, and 3% PBST was used as a blocking buffer. Rabbit anti-pig IgG-HRP was used as a secondary antibody (1:35,000). After a 1-h incubation at room temperature on a plate shaker, the plate was washed with 0.05% PBST, 3,3ʹ,5,5-tetramethylbenzidine (Invitrogen Canada Inc., Burlington, ON, Canada) was added, incubated on a plate shaker for 15 min at room temperature in the dark, then 2N H_2_SO_4_ was used as the stop solution. Samples were analyzed in duplicate, and optical density was measured at 450 nm with a reference wavelength of 570 nm using the Epoch 2 microplate spectrophotometer (BioTek Instruments Inc., Winooski, VT). This protocol was followed for both IgA and IgM with corresponding primary (1:40,000 and 1:35,000, respectively) and secondary (1:55,000 and 1:55,000, respectively) antibodies, and samples were diluted 1:1,000 and 1:500 for IgA and IgM analyses, respectively. Initial test plates were conducted using pooled gilt, piglet, and colostrum samples, respectively, to determine optimal capture and detection of antibody concentrations and sample dilutions. Standard curves were generated using purified anti-pig IgA, IgG, and IgM (Alpha Diagnostic International, San Antonia, TX). The average intra- and inter-assay variations were 4.32%, 4.61%, 2.51% and 3.43%, 3.44%, 4.01% for IgA, IgM, and IgG, respectively.

Fecal samples were thawed, and short-chain fatty acid (**SCFA**) and branched-chain fatty acid (**BCFA**) were analyzed according to Leung et al. (2018) using high-performance liquid chromatography. Gilt plasma samples were also sent to the Animal Health Laboratories (University of Guelph, Guelph, ON, Canada) for analysis of plasma minerals, proteins, and metabolites.

### Calculations and statistical analysis

Data from the study were analyzed using the GLIMMIX procedure in SAS (University Edition; SAS Inst. Inc., Cary, NC). Treatment was the main effect and block (breeding batch) was considered a random effect. Initial gilt BW on day 85 of gestation was used as a covariate for sow performance outcomes and time (minutes) between birth of the first piglet and colostrum sampling was used as a covariate for day 1 Ig in gilt and piglet plasma and in colostrum. Orthogonal coefficients produced via the IML procedure were used to construct linear, quadratic, cubic, and quartic contrast statements of the unequally spaced dietary inclusion levels of HY. Probability (*P*) values of <0.05 were considered significant and 0.05 ≤ *P ≤ *0.10 were considered tendencies.

## Results

Analyzed chemical compositions of the experimental diets (Table 1) were consistent with calculated values for crude protein but were on average, 15% and 12% lower than calculated values for calcium and phosphorus, respectively. One gilt was removed from each of the HY0.25, HY0.5, and HY1.0 dietary treatment groups due to farrowing complications and/or lameness and the data were excluded from statistical analysis.

### Gilt and piglet performance

Gilt BW on days 85 and 110 of gestation and on lactation day 1, gilt BW change in gestation, change in lactation, and average daily feed intake in gestation were not influenced by HY inclusion level (*P* > 0.10; Table 2). Gilt BW on lactation day 19 tended to be influenced by HY inclusion level (cubic; *P *= 0.097) and gilt average daily feed intake in lactation tended to increase then decrease with increasing HY inclusion (quadratic; *P *= 0.085).

**Table 2.** Gilt body weight and feed intake during late gestation and lactation

	Treatments^1^						*P*-values^2^			
	HY0	HY0.25	HY0.5	HY1.0	HY1.2	SEM3	Linear	Quadratic	Cubic	Quartic
No.^4^	10	9	9	9	10					
*Gilt BW, kg*										
Gestation day 85	191.3	191.1	189.7	190.6	191.4	4.5	0.999	0.779	0.934	0.881
Gestation day 110	209.7	210.5	211.8	211.3	210.5	4.2	0.873	0.733	0.946	0.919
Lactation d 1	189.6	193.8	195.6	193.5	192.9	5.4	0.631	0.660	0.893	0.241
Lactation d 19	180.1	190.5	187.8	176.7	184.1	5.7	0.661	0.874	0.097	0.323
Change in gestation BW	18.5	19.	22.2	20.8	20.5	2.6	0.548	0.432	0.999	0.598
Change in lactation BW	−9.4	−3.3	−6.8	−16.8	−8.8	5.3	0.343	0.782	0.103	0.889
*Gilt average daily feed intake, kg*										
Gestation	2.59	2.60	2.58	2.60	2.60	0.01	0.327	0.366	0.973	0.177
Lactation	4.53	5.54	5.03	5.01	4.79	0.31	0.917	0.085	0.270	0.120

^1^Dietary treatments: HY0, control gestation and lactation diets; HY0.25, HY0.5, HY1.0, and HY1.2 included either 0.25%, 0.5%, 1.0%, or 1.2%, respectively, of yeast inactivated via hydrolyzation. Diets were fed between day 85 of gestation and day 19 of lactation (weaning).

^2^ *P*-values for linear, quadratic, cubic, and quartic contrast statements.

^3^Maximum value for the standard error of the means.

^4^Number of gilts evaluated.

There were no treatment effects on the total number of piglets born or born alive, litter size at weaning, preweaning piglet mortality, litter or piglet birth weights, the proportion of low-birth-weight (<1.0 kg) piglets or litter weight at weaning (*P* > 0.10; Table 3). Litter size after cross-fostering was influenced by maternal dietary HY inclusion (quartic; *P* < 0.05). Piglet ADG during lactation (linear, quadratic, and quartic; *P *< 0.05) and BW at weaning (quadratic and quartic; *P *< 0.01) increased then decreased with increasing maternal dietary HY inclusion.

**Table 3.** Litter characteristics and growth performance during the lactation period

	Treatments^1^						*P*-values^2^			
	HY0	HY0.25	HY0.5	HY1.0	HY1.2	SEM^3^	Linear	Quadratic	Cubic	Quartic
No.^4^	10	9	9	9	10					
*Litter characteristics*										
Total born, *n*	12.7	13.4	13.5	13.0	13.4	0.9	0.774	0.649	0.571	0.932
Born alive, *n*	11.8	12.3	12.8	12.5	12.6	0.9	0.548	0.561	0.830	0.816
Litter size post-cross fostering, *n*	12.1	11.6	12.4	11.9	12.3	0.3	0.403	0.867	0.763	0.026
Proportion of low-birth-weight piglets, %^5^	9.61	13.79	7.16	10.19	14.59	4.65	0.581	0.465	0.504	0.539
Litter size at weaning, *n*	11.3	10.5	11.1	11.0	11.9	0.4	0.161	0.101	0.971	0.148
Preweaning mortality, %	6.20	8.99	10.04	7.21	3.27	3.58	0.452	0.198	0.910	0.958
*Growth performance* ^6^										
Litter birth weight, kg	17.12	17.88	18.81	18.23	18.41	1.08	0.432	0.432	0.786	0.687
Piglet birth weight, kg	1.41	1.39	1.40	1.40	1.40	0.04	0.972	0.837	0.806	0.814
Litter wean weight, kg	66.10	65.80	67.69	71.70	69.20	3.84	0.256	0.888	0.475	0.922
Piglet body weight at weaning, kg	5.74	6.39	6.03	6.29	5.88	0.11	0.651	0.007	0.990	<0.001
Average daily gain, g	226	254	233	240	217	56	0.048	<0.001	0.779	<0.001

^1^Dietary treatments: HY0, control gestation and lactation diets; HY0.25, HY0.5, HY1.0, and HY1.2 included either 0.25%, 0.5%, 1.0%, or 1.2%, respectively, of yeast inactivated via hydrolyzation. Diets were fed between day 85 of gestation and day 19 of lactation (weaning).

^2^ *P*-values for linear, quadratic, cubic, and quartic contrast statements.

^3^Maximum value for the standard error of the means.

^4^Number of litters evaluated.

^5^Piglets born weighing <1.0 kg were considered low-birth-weight piglets.

^6^Growth performance metrics calculated using post-cross fostering piglet body weights.

### Plasma and colostrum immunoglobulins

On day 1 of lactation, there were no treatment effects on plasma or colostrum concentrations of immunoglobulins (IgA, IgG, and IgM) in gilts (*P* > 0.10; Table 4). On day 1 of lactation, piglet plasma IgA (linear and cubic; *P *= 0.086 and *P *< 0.05, respectively) and IgM (linear and cubic; *P *< 0.05) concentrations increased with increasing maternal dietary HY inclusion, but piglet plasma concentrations of IgG were not affected by dietary treatment. On day 19 of lactation, gilt plasma IgG and IgM concentrations were not affected by dietary treatment (*P* > 0.10), but plasma IgA concentration tended to increase then decrease with HY inclusion (quadratic; *P *= 0.096). On day 19 of lactation, piglet plasma IgG concentration tended to increase with increasing maternal dietary inclusion of HY (linear*; P *=* *0.099) but plasma concentrations of IgA and IgM were not affected by dietary treatment (*P* > 0.10).

**Table 4.** Plasma and colostrum immunoglobulin A, G, and M concentrations 24-h after farrowing and at weaning

	Treatments^1^						*P*-values^2^			
	HY0	HY0.25	HY0.5	HY1.0	HY1.2	SEM^3^	Linear	Quadratic	Cubic	Quartic
No.^4^	10	9	9	9	10					
Immunoglobulin (Ig) concentrations										
Day 1^5^										
*Gilt plasma*										
IgG, ug/mL	1,073	1,077	1,078	1,069	1,075	7	0.770	0.771	0.472	0.686
IgA, pg/mL	108	107	109	109	109	1	0.260	0.943	0.376	0.122
IgM, ng/mL	64	60	62	60	61	2	0.376	0.385	0.525	0.220
*Colostrum*										
IgG, ug/mL	984	990	1,017	1,002	992	18	0.690	0.192	0.795	0.482
IgA, pg/mL	106	104	109	108	108	4	0.397	0.674	0.666	0.419
IgM, ng/mL	42	46	48	44	45	5	0.994	0.309	0.437	0.816
*Piglet plasma*										
IgG,ug/mL	1,110	1,100	1,103	1,116	1,118	15	0.243	0.345	0.521	0.921
IgA, pg/mL	118	116	118	121	119	2	0.086	0.451	0.024	0.655
IgM, ng/mL	60	55	58	63	61	3	0.040	0.306	0.025	0.597
Day 19										
*Gilt plasma*										
IgG, ug/mL	1,066	1,065	1,064	1,056	1,053	10	0.251	0.807	0.988	0.817
IgA, pg/mL	104	105	107	107	106	2	0.167	0.096	0.760	0.731
IgM, ng/mL	56	54	51	51	52	2	0.120	0.123	0.833	0.652
*Piglet plasma*										
IgG, ug/mL	1,020	1,014	1,014	1,025	1,029	9	0.099	0.260	0.719	0.954
IgA, pg/mL	93	96	96	97	97	1	0.109	0.283	0.523	0.413
IgM, ng/mL	36	36	38	35	38	3	0.605	0.991	0.121	0.139

^1^Dietary treatments: HY0, control gestation and lactation diets; HY0.25, HY0.5, HY1.0, and HY1.2 included either 0.25%, 0.5%, 1.0%, or 1.2%, respectively, of yeast inactivated via hydrolyzation. Diets were fed between day 85 of gestation and day 19 of lactation (weaning).

^2^ *P*-values for linear, quadratic, cubic, and quartic contrast statements.

^3^Maximum value for the standard error of the means.

^4^Number of gilts and litters evaluated; each litter had 2 median body weight piglets (1 barrow, 1 gilt) sampled.

^5^Time (minutes) between birth of the first piglet and sampling was used as a covariate for day 1 immunoglobulin concentrations for plasma (piglet and sow) and colostrum samples.

### Gilt fecal short-chain and branched-chain fatty acids and plasma biochemistry at weaning

There were no treatment effects on the concentrations of fecal acetic, butyric, formic, lactic, propionic, or iso-butyric acid concentrations in gilts at weaning (Table 5). Fecal valeric acid tended to increase then decrease (quadratic; *P *= 0.074) and iso-valeric acid tended to increase (linear; *P *= 0.080) with increasing dietary HY inclusion.

**Table 5.** Gilt fecal short and branched-chain fatty acids at weaning

	Treatments^1^						*P*-values^2^			
	HY0	HY0.25	HY0.5	HY1.0	HY1.2	SEM^3^	Linear	Quadratic	Cubic	Quartic
No.^4^	10	9	9	9	10					
*Short-chain fatty acid, µmol/mL*										
Acetic	6.91	9.87	7.58	6.15	6.26	0.78	0.350	0.388	0.938	0.454
Butyric	4.01	4.17	4.25	4.03	3.82	0.25	0.420	0.243	0.976	0.965
Formic	0.82	1.06	0.83	1.10	0.69	0.25	0.709	0.420	0.488	0.205
Lactic	0.76	0.63	0.84	0.53	0.61	0.12	0.199	0.700	0.810	0.101
Propionic	2.32	2.52	2.49	2.13	1.96	0.25	0.108	0.240	0.740	0.975
Valeric	0.27	0.33	0.35	0.36	0.31	0.03	0.599	0.074	0.518	0.740
*Branched-chain fatty acids, µmol/mL*										
Iso-butyric	2.02	2.22	2.26	2.14	2.13	0.11	0.842	0.122	0.485	0.941
Iso-valeric	0.40	0.46	0.45	0.38	0.34	0.05	0.080	0.108	0.667	0.979

^1^Dietary treatments: HY0, control gestation and lactation diets; HY0.25, HY0.5, HY1.0, and HY1.2 included either 0.25%, 0.5%, 1.0%, or 1.2%, respectively, of yeast inactivated via hydrolyzation. Diets were fed between day 85 of gestation and day 19 of lactation (weaning).

^2^ *P*-values for linear, quadratic, cubic, and quartic contrast statements.

^3^Maximum value for the standard error of the means.

^4^Number of gilts evaluated.

At weaning, gilt plasma concentrations of sodium and calculated osmolality increased then decreased with increasing dietary HY inclusion (quadratic; *P *< 0.05) and plasma concentrations of chlorine tended to increase with HY inclusion (linear, quadratic, and quartic; *P *= 0.066, *P *= 0.069, and *P *= 0.046, respectively), whereas other minerals, total protein, albumin, and globulin concentrations were not influenced by dietary treatment (Table 6). Plasma glucose and total bilirubin concentrations tended to decrease with HY inclusion (linear; *P *= 0.067 and *P *= 0.087, respectively) and plasma haptoglobin tended to be influenced by HY inclusion (quartic; *P *= 0.086), but plasma urea and β-hydroxybutyrate were unaffected by dietary treatment.

**Table 6.** Plasma biochemistry at weaning for gilts at weaning

	Treatments^1^						*P*-values^2^			
	HY0	HY0.25	HY0.5	HY1.0	HY1.2	SEM^3^	Linear	Quadratic	Cubic	Quartic
No.^4^	10	9	9	9	10					
*Minerals, mmol/L*										
Sodium	140	142	142	143	141	1	0.194	0.026	0.266	0.105
Chlorine	95	97	97	99	96	1	0.066	0.069	0.354	0.046
Potassium	4.31	4.53	4.45	4.25	4.44	0.17	0.839	0.660	0.169	0.975
Calcium	2.50	2.50	2.48	2.52	2.42	0.44	0.376	0.460	0.184	0.331
Phosphorus	2.17	2.36	2.35	2.20	2.23	0.95	0.683	0.136	0.199	0.914
Magnesium	0.74	0.78	0.77	0.81	0.77	0.26	0.203	0.263	0.620	0.262
Calculated osmolality, mmol/L	279	281	282	284	279	1	0.312	0.037	0.138	0.221
*Protein, g/L*										
Total protein	69	70	70	69	71	2	0.812	0.977	0.397	0.981
Albumin	43	41	43	43	43	1	0.392	0.762	0.201	0.452
Globulin	26	29	27	25	28	2	0.852	0.906	0.153	0.732
*Metabolites, mmol/L*										
Urea	4.39	3.95	4.39	5.06	4.42	0.47	0.312	0.986	0.118	0.943
Glucose	5.81	5.12	5.53	5.07	4.81	1.64	0.067	0.979	0.377	0.296
Total bilirubin	1.75	1.10	0.90	0.82	0.80	0.41	0.087	0.286	0.676	0.901
Haptoglobin	2.72	3.73	3.21	3.39	3.29	0.40	0.402	0.158	0.231	0.086
β-hydroxybutyrate, µmol/L	18	17	15	16	19	3	0.781	0.141	0.736	0.841

^1^Dietary treatments: HY0, control gestation and lactation diets; HY0.25, HY0.5, HY1.0, and HY1.2 included either 0.25%, 0.5%, 1.0%, or 1.2%, respectively, of yeast inactivated via hydrolyzation. Diets were fed between day 85 of gestation and day 19 of lactation (weaning).

^2^ *P*-values for linear, quadratic, cubic, and quartic contrast statements.

^3^Maximum value for the standard error of the means.

^4^Number of gilts evaluated.

## Discussion

The objective of the current study was to assess the dose–response of HY inclusion in the maternal diet on offspring growth performance and Ig transfer prior to weaning in pigs. Improvements in preweaning growth performance and piglet BW at weaning with increasing inclusion levels of HY in the late gestation and lactation diets of gilts corresponded to improvements in piglet plasma concentrations of IgA and IgM 24 h after birth and plasma IgG concentrations at weaning. Therefore, it appears that maternal intake of HY improved the transfer of Ig to the offspring, which had preweaning growth-promoting effects. In addition, it is noted that the relationships between piglet growth performance and HY inclusion in the maternal diet were quadratic, indicating that at high inclusion levels of HY, offspring growth performance was negatively affected. Therefore, an optimal inclusion level of HY in the maternal diet can be anticipated at around 0.25% for piglet growth performance outcomes, but greater passive immune transfer could be achieved at higher maternal dietary HY inclusion levels.

Previous work has also demonstrated improvements in the preweaning growth performance of the offspring when yeast products were provided to sows during gestation and lactation (Chance et al., 2022); however, some studies found no differences in the weaning weight of offspring when sow diets were supplemented with yeast (Jang et al., 2013; Hasan et al., 2018; Peng et al., 2020). Yeast additives come in a wide variety (i.e., pre-, pro-, or postbiotics), and the yeast form used seems to have a substantial impact on its efficacy. For example, Chance et al. (2022) found positive effects on offspring growth using a blend of yeast pre- and probiotics whereas feeding only yeast prebiotics did not elicit positive effects on offspring growth performance (Jang et al., 2013; Peng et al., 2020). Moreover, since yeast is an immune-modulating additive, it is possible that at high inclusion levels (e.g., >1.0%) it may overstimulate the immune system leading to a negative growth response (Hasan et al., 2018). Indeed, in the current study, gilt plasma haptoglobin and lactation day 1 piglet Ig were affected by HY inclusion, which indicates HY activated the gilt’s immune system to some degree. The precise mechanism of reduced piglet growth performance at high yeast inclusion levels in the maternal diet is unknown. It can be postulated that over-activation of the immune system resulted in increased maintenance energy requirements for the gilt, and thus, a possible reduction in milk production. Alternatively, yeast products could interact with other components of the diet, reducing feed intake and nutrient digestibility. In the current study, however, gilt performance outcomes (i.e., BW loss) were not negatively affected at high HY inclusion levels. Conversely, although gilts were fed in a raised feeder, it is possible that piglets sampled the lactation diet, which could directly impact piglet growth; however, piglet feed intake was not recorded. Further work is required to assess why a negative response in offspring preweaning growth performance was observed with high inclusion levels of HY in the maternal diet, but the current findings demonstrate the importance of dose–response studies to determine inclusion levels that maximize perceived benefits without negative outcomes.

Yeast is a favorable additive for pigs, since it is immune modulating, specifically through the actions of β-glucans and mannan oligosaccharides (Kogan and Kocher, 2007; Kiarie et al., 2011; Christensen et al., 2022). β-glucans are a type of polysaccharide that can interact with intestinal epithelium, macrophages, neutrophils, and dendritic cells (Raa, 2015). The activation of these cell types leads to an improved innate immune response (i.e., improved antibody production, faster tissue repair, reduced enteric infections, and reduced toxicity of bacterial endotoxins; Kogan and Kocher 2007; Raa, 2015). It is through the action of β-glucans that yeast increases the production of lysozymes, antimicrobial peptides, and immunoglobulins (Stuyven et al., 2009). The improvement of day 1 IgA and IgM plasma concentration in the piglets suggests that HY was effective in eliciting the immune response that is expected with β-glucans (Raa, 2015) and that maternal transfer was correspondingly improved. By weaning, however, differences in plasma Ig concentrations were less apparent.

Yeast also contains mannan oligosaccharides which are another important component that contributes to the immunomodulating properties of yeast (Kogan and Kocher 2007). Mannan oligosaccharides bind to bacterial cell walls such as *Escherichia coli* preventing adherence to the intestinal wall where they can proliferate; instead, bacteria are flushed through the gastrointestinal tract, leaving the pig unaffected (Kogan and Kocher 2007). The presence of *E. coli* in the intestine was not measured in the sow or piglets in the current study; however, this mechanism is expected to provide protection at weaning if piglets were exposed to the lactation diet prior to weaning. Through birth, suckling, and housing, the sow microbiome is passed to the offspring (Hasan et al., 2018). Previous work has shown that yeast supplementation in sow diets can positively impact the sow microbiome (i.e., increase the abundance of *Roseburia* and lower abundance of *Desulfovibrio*) and can increase the abundance of specific taxa that were associated with improved preweaning piglet growth (Hasan et al., 2018). In the current study, there were minimal differences in gilt fecal SCFA and BCFA; however, these are not robust indicators of the microbiome. Although previous studies have reported that yeast in sow diets can affect the offspring microbiome, further work needs to be done to assess carryover effects for the offspring after weaning.

In addition to the HY effect on immune transfer from sow to offspring, the current study showed that HY also elicited some positive effects for the sow. The immune-modulating function of yeast also includes the antioxidant activities of β-glucans (Boontiam et al., 2020). Indeed, in the current study, gilt plasma bilirubin concentrations decreased with increasing dietary HY inclusion, suggesting improved liver function, likely through increased antioxidant capacity (Liu et al., 2017). Other studies feeding yeast additives have also found that oxidative stress markers (i.e., superoxide dismutase, malondialdehyde, and catalase) were improved when yeast was fed to nursery pigs (Liu et al., 2017). In reproductive sows, oxidative stress increases as gestation progresses due to rapid cellular growth and differentiation (Tobola-Wróbel et al., 2020) and continues throughout lactation due to high rates of protein and fat oxidation (Al-Gubory et al., 2010; Berchieri-Ronchi et al., 2011). Therefore, further work should be done to confirm if HY can improve the antioxidant capacity of sows.

In conclusion, in this study the HY additive that was supplemented to sow diets proved to be effective in improving preweaning offspring growth performance and Ig transfer. Further work should be conducted to determine how the offspring from HY-supplemented gilts perform after weaning.

## Abbreviations

BCFA: branched-chain fatty acids
BW: body weight
HY: inactivated yeast via hydrolyzation
HY0: HY included in diets at 0%
HY0.25: HY included in diets at 0.25%
HY0.5: HY included in diets at 0.5%
HY1.0: HY included in diets at 1.0%
HY1.2: HY included in diets at 1.2%
Ig: immunoglobulin
P: probability
PBST: 0.05% phosphate-buffered saline with tween-20
SCFA: short-chain fatty acids
SID: standardized ileal digestible
